# OVAS: an open-source variant analysis suite with inheritance modelling

**DOI:** 10.1186/s12859-018-2030-8

**Published:** 2018-02-08

**Authors:** Monika Mozere, Mehmet Tekman, Jameela Kari, Detlef Bockenhauer, Robert Kleta, Horia Stanescu

**Affiliations:** 1 Division of Medicine, University College London, London, NW3 2PF UK; 2 Pediatric Nephrology Center of Excellence and Pediatric Department, Faculty of Medicine, King Abdulaziz University, Jeddah, Kingdom of Saudi Arabia

**Keywords:** Open source, Variant analysis, Inheritance model, Mosaic, Bootable, Live environment

## Abstract

**Background:**

The advent of modern high-throughput genetics continually broadens the gap between the rising volume of sequencing data, and the tools required to process them. The need to pinpoint a small subset of functionally important variants has now shifted towards identifying the critical differences between normal variants and disease-causing ones. The ever-increasing reliance on cloud-based services for sequence analysis and the non-transparent methods they utilize has prompted the need for more in-situ services that can provide a safer and more accessible environment to process patient data, especially in circumstances where continuous internet usage is limited.

**Results:**

To address these issues, we herein propose our standalone Open-source Variant Analysis Sequencing *(OVAS)* pipeline; consisting of three key stages of processing that pertain to the separate modes of annotation, filtering, and interpretation. Core annotation performs variant-mapping to gene-isoforms at the exon/intron level, append functional data pertaining the type of variant mutation, and determine hetero/homozygosity. An extensive inheritance-modelling module in conjunction with 11 other filtering components can be used in sequence ranging from single quality control to multi-file penetrance model specifics such as X-linked recessive or mosaicism. Depending on the type of interpretation required, additional annotation is performed to identify organ specificity through gene expression and protein domains. In the course of this paper we analysed an autosomal recessive case study. OVAS made effective use of the filtering modules to recapitulate the results of the study by identifying the prescribed compound-heterozygous disease pattern from exome-capture sequence input samples.

**Conclusion:**

OVAS is an offline open-source modular-driven analysis environment designed to annotate and extract useful variants from Variant Call Format (VCF) files, and process them under an inheritance context through a top-down filtering schema of swappable modules, run entirely off a live bootable medium and accessed locally through a web-browser.

**Electronic supplementary material:**

The online version of this article (10.1186/s12859-018-2030-8) contains supplementary material, which is available to authorized users.

## Background

The technological evolution of sequencing platforms has progressed rapidly since the completion of the Human Genome project via Sanger sequencing methods [14, 20]. Modern high-throughput sequencing (HTS) approaches post-Sanger era have superseded this standard, allowing for a greater number of variants to be sequenced across the whole genome by employing powerful mass fragmentation/amplification approaches upon a target sequence [2, 16].

The raw sequence FASTQ reads produced by these HTS platforms are aligned to a specific version of the NCBI reference sequence and collated into a Binary Alignment Map (BAM) where variants of interest can then be individually “called” to form a Variant Call Format (VCF) file of novel or known variants conforming to a specific variant database (dbSNP) [5, 17].

BAM and VCF data are orthogonally related, with the former storing horizontal stretches of FASTA sequence reads aligned unevenly on top of one another forming “pile ups”, and the latter taking vertical cross-sections of these pileups at specific loci to form a variant call.

The VCF specification was designed for the 1000 Genomes project to produce a robust format that could house the many samples often sequenced under the same batch, but has since been adopted by projects such as UK10K, dbSNP, NHLBI Exome Project, amongst others. The format is flexible with annotations, where additional fields can be outlined in the header and adhered to in the body of the data. Each line of the VCF body describes a single variant; physical position paired with a reference allele (as ascribed by a reference genome consistent across the entire VCF file) and alternate alleles that appear within samples. Major and minor alleles are specific only to the sample population but their frequencies can be pre-computed and appended to a variant line as additional information to then be utilized in small population analyses such as inheritance modelling [5].

Variant analysis suites all work under the same principle; filtering variants under a user-specified set of criteria against the various variant annotations present in the VCF in order to produce a subset informative to the phenotype. Stringent filtering measures will produce a smaller set with the drawback of missing key causative variants, and more optimistic filtering measures will produce too many false positives. The effectiveness of an analysis rests primarily upon the accuracy of the variant annotations which can attribute to as much as 15% of false negatives [22], as well as the frequency of false negatives that are discarded due to overly-stringent quality filtering. A common approach to addressing both issues is through learning algorithms that can be trained to favour individual variants over others with the caveat of producing results via ‘black-box’ methods that may create some disparity between the user and their data [18].

A more transparent approach is to expand the scope of the filtering beyond the variant/gene-level and explore variants under a larger trait-penetrance context.

Mendelian traits conform to the four classical modes on inheritance of autosomal/X-linked, dominant/recessive penetrance. Dominant disorders result from the inheritance of a single mutant allele which is manifested in each subsequent generation with a 50% chance of likelihood in offspring from a single affected parent. Recessive traits require the inheritance of two mutant alleles on opposing strands in order to block any functioning copies of the causative gene. Parents are typically carriers with affected offspring. These disorders are at times a result of consanguineous marriages, where a single mutant allele manifests on both alleles due to the multiple paths of descent it can undertake [10]. In the case of X-linked recessive inheritance, males with a single mutant copy are hemizygous and must express the phenotype.

For non-Mendelian disorders, we also consider the special case of *mosaicism*; where de novo mutations produce two or more populations of cells that result in segregated sets of genotypes within the same individual. Mosaic genotypes can be revealed stochastically by measuring alternate allele frequencies against expected values [1].

Here we outline our Open-source Variant Analysis Suite (OVAS) that makes use of these inheritance modelling scenarios with the aim to vastly reduce the number of false positives.

## Implementation

The core ideology behind OVAS was to preserve the VCF specification at each step of the analysis, and this is catered to extensively within the pipeline where each module inputs and outputs VCF file(s) in order to facilitate the chaining of subsequent pipeline modules downstream. This allows for full analysis transparency, where results can be extracted at any stage of an ongoing analysis.

Module ordering is flexible in this regard, with the exception of the primary annotation modules which are required to run prior to any filtering in order to produce an effective analysis of the variants. Pre-existing gene and function annotations within input data are ignored unless generated by a previous run of the OVAS pipeline, supplanting foreign annotations with the pipeline’s own if required. This is to ensure unambiguous results stemming from external annotations using unknown sources that may result in erroneous output variants.

OVAS annotates variants using data from trusted public domain databases such as RefGene, dbSNP, UniProt, and many others through the UCSC Genome Browser’s MySQL back-end portal [11]. The explicitly open nature of pipeline also prompts a predilection towards open-source or scripted languages and frameworks, which further serve to uphold the confidence between the end-user and their data.

**Fig. 1:**
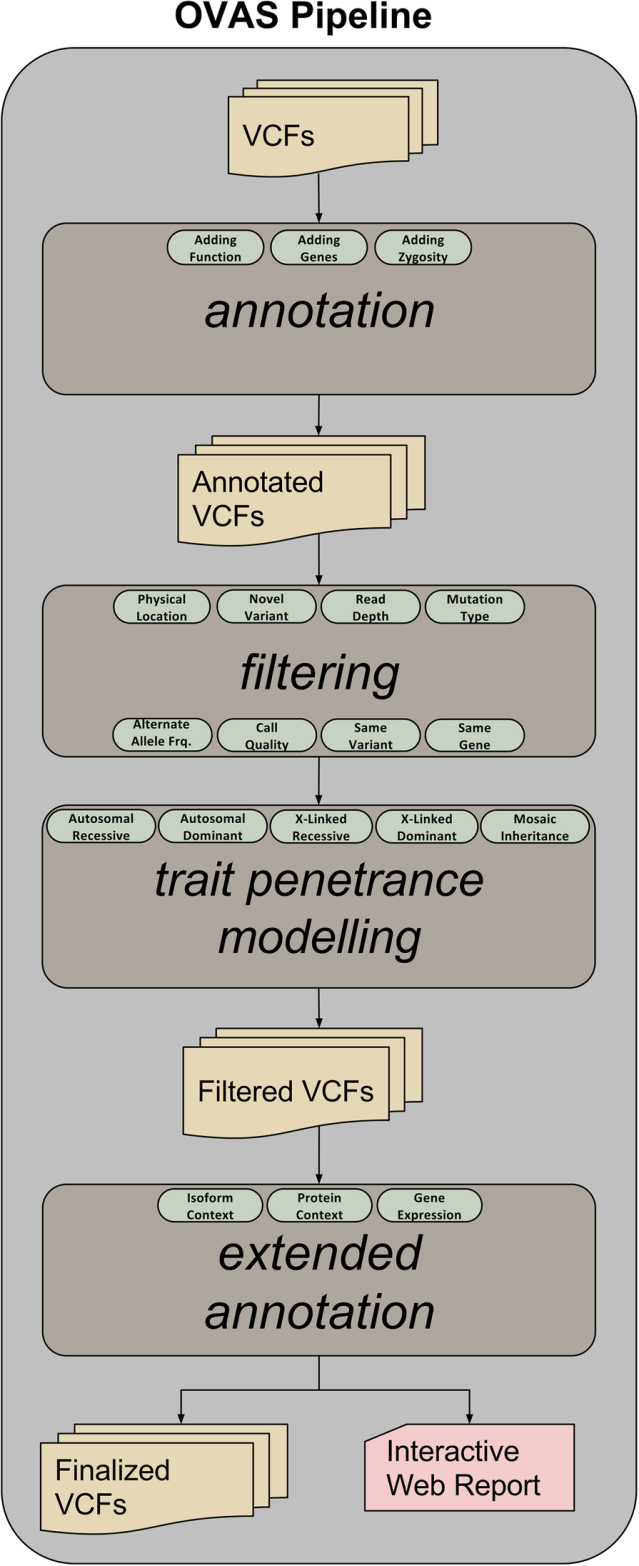
Overall structure of the OVAS pipeline: VCF files as referenced by a pedigree file are fed into the pipeline and are processed in turn by the core annotation, optional filtering, trait penetrance modelling, and additional annotation modules

Core pipeline functionality is managed through back-end shell scripts which serve to chain subsequent pipeline modules as shown in Fig. 1. The modular-centric design and development enables each pipeline module to be run as a standalone script without the need for an overarching framework. It also allows for the pipeline to be initiated manually for the more commandline-oriented users, where input VCF files can be placed into a new folder on the desktop along with a pedigree file and an appropriate configuration file (see manual in software repository), and executed via the starting script.

**Fig. 2:**
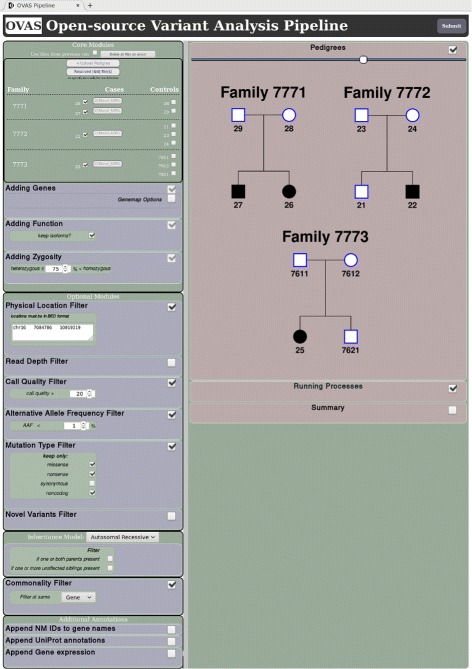
Web-interface displaying an ongoing analysis. The left sidebar shows the user-set configurations, and the central-right box displays the pedigrees used in the analysis stacked above a real-time progress box. Once complete, a summary will automatically open in a new browser tab. Here, 4 individuals’ data from 3 families were analysed, with pipeline settings configured in the left side-bar; case VCF files auto selected, core modules running on default settings, optional modules configured to use linkage data, call quality filtering (> 20), rare variant filtering (< 1*%*), non-synonymous mutations requested, and an autosomal recessive inheritance filtering model applied in conjunction with gene-level variant filtering

However, OVAS was designed to cater towards all users, and is accessed primarily through a graphical user-interface within a web browser which facilitates in the VCF file placement and configuration process through file selection dialogues and configurable forms to generate run profiles, as well a means to manage and view ongoing analyses as shown in Fig. 2.

OVAS is split into three separable parts, with each component encapsulated by the next; the processing back-end, the web-interface front-end, and the live operating system. Instructions to acquire and set up each as distinct items are provided in the software repository, but OVAS is bundled principally as an all-encompassing standalone bootable ISO image that can be deployed onto a DVD or USB.

### Pipeline overview

OVAS is composed of five main stages of processing followed by a generated report detailing the findings of the analysis.

#### Pre-processing

All VCF files immediately undergo initial preparation upon file submission from the web interface, where a background shell script renames the files to better emulate their pedigree counterparts, and asserts that all variants are in correct order following a chromosome:position sorting scheme.

#### Core annotation

The annotation stages of the pipeline then affix the variants with the relevant metadata to aid in the filtering process against user-specified criterion throughout the rest of the pipeline.

First, a gene context is appended to the variants specific to a level of detail preferred by the user. This includes, but is not limited to; exons, introns, (donor/acceptor) splice sites, (5’/3’) UTR, and (default 500bp) upstream/downstream promoters. Wholly intergenic regions are discarded by default, which often results in a vast majority of initial variants being filtered out (approximately 90% for whole-genome sequence data).

Ensuing functional changes and the resulting mutation types (synonymous, missense, nonsense, etc) are also annotated to the variant by performing cDNA lookups of the variant against reference genome FASTA data and determining the subsequent changes at the codon and amino-acid level for all sense and anti-sense gene transcripts.

The VCF specification generally denotes a single variant per line and OVAS vehemently upholds this policy when a variant bisects multiple gene transcripts. This is notably different from UCSC’s Variant Annotator [8], which despite taking in VCF input, does not preserve the format and reports multiple bisecting sites upon adjacent lines. For a given variant, OVAS ensures that each gene context and correlating functional change are stored in-line as separate associative arrays that are indexed to the same gene transcript.

Finally, heterozygosity and homozygosity are assigned to the variant based on nucleotide base count alone, addressing a confidence issue in the zygosity assignment provided by pre-processed variants.

#### Filtering

Once fully annotated, variants are then subject to the conventional filtration modules that act upon the standard positional and INFO fields provided by VCF data against regions/thresholds set by the user. Specifically; *Physical Location Filter*, *Novel Variation Filter*, *Read Depth Filter*, and *Call Quality Filter*.

OVAS provides a *Mutation Type Filter* which acts upon the functional annotations provided by OVAS to keep/discard any variation of missense, nonsense, and synonymous mutations. It also provides an *Alternate Allele Frequency* module which screens for rarity by comparing alternate allele frequencies against the reference genome via dbSNP (version 147).

Variants are also filtered over multiple VCF files, with the *Same Variant Filter* discarding variants not shared across all cases, and the *Same Gene Filter* discarding those that do not reside within the same gene context shared across all cases. Both modules are used extensively in the inheritance filters.

#### Inheritance filtering

This section performs trait penetrance modelling for differently affected individuals following sibling-sibling, and sibling-parent relations. For all detected parent-offspring trios, variants undergo context-based filtering depending on the penetrance-model specified:

##### Autosomal dominant

The phenotype is caused by a single mutant autosomal allele, and affected individuals must have affected parents, mapping any {HOM,HET} ↦{HET,HOM} under complete penetrance. Under a *de novo* context all common affected variants are filtered against unaffected controls, otherwise variant commonality is kept within sibling groups.

##### Autosomal recessive

The phenotype is caused by a loss of function stemming from both copies of an autosomal gene, at times from the result of consanguineous breeding. Two paths of transmission are considered from parent ↦offspring depending on whether the affected offspring variant is compound-heterozygous (C-HET) or homozygous (HOM). Under the assumption that parents are carriers:

1. **HOM**, Both parents transmit a single HET variant which manifests as a single HOM variant in the offspring, i.e. {HET/HET} ↦HOM.
2. **C-HET**, Parents are carriers for different HET variants across a common gene, which compound in offspring as multiple HET variants within said gene. If HET1 and HET2 are distinct variants within the same gene from different parents, then this can be represented under a gene context as {HET1/HET2} ↦ {HET1+HET2} mapping to produce a C-HET gene.

Siblings are then filtered for common variants existing within affecteds siblings only, discarding those that are homozygous in unaffected controls.

#### X-linked dominant

As with autosomal dominant but with the mutant allele on the X-chromosome.

#### X-linked recessive

As with autosomal recessive but with mutations occurring on the X-chromosome. Males with a single mutant copy are hemizygous and are treated as homozygous, exempting them from compound heterozygosity checking.

#### Mosaicism

Mosaic inheritance is treated as a special case, where allele frequencies are pre-calculated for each variant and then filtered against user-set thresholds conforming to expected mosaic frequency ranges (typically between 10–35%).

#### Extended annotation

The last processing stage of pipeline constitutes a small set of potentially causative variants that successfully passed through the main filtering stages and require finer annotation and analysis that was too costly to perform for all variants at the start. Here, gene transcripts are assigned RefSeq IDs to better distinguish them against external sources(*Isoform Context*), variants falling within known protein domains provided by UniProt are further functionally annotated (*Protein Context*), and tissue-specific data from the Encode GNF Atlas2 database are used to filter for/against genes falling within user-specified gene expression thresholds (*Gene Expression*).

#### Web report

**Fig. 3:**
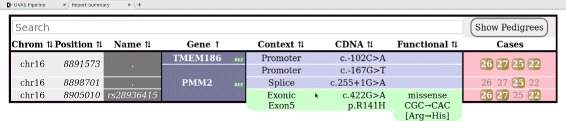
The summary tab contains a comprehensive report of potential causative variants discovered in the analysis. The report is interactive and can perform dynamic filtering and sorting upon any data field. Columns containing adjacent data in the rows above or below are merged for conciseness. Toggling the column headers sorts the data in that field in ascending/descending order, and the search bar can be used to isolate variants of interest such as those which cause missense mutations, or variants existing in promoter regions. Gene isoforms can be filtered in or out by using the “ISO” or “REF” keyword, respectively. Pedigrees can be quickly viewed by hovering over the *Show Pedigrees* button above the *Cases* and *Controls* column headers, each of which display the presence and zygosity of the variant in sample individuals, with striped colouring for heterozygous and solid colouring for homozygous. Presented are the same 4 individuals from Fig. 2, showing compound-heterozygous mutations in *PMM2*. Note, the promoter mutation is located within a bidirectional promoter region (i.e. *PMM2/TMEM186*)

All remaining variants across all output VCF files are then consolidated into an interactive HTML table which summarizes variants under sortable and filterable columns of chromosome, position, rsID, gene, gene context, cDNA and protein change, functional change, and heterozygous/homozygous occurrence in cases and controls (see Fig. 3).

This provides a good overview of potentially causative variants, especially in recessive disease models where compound-heterozygosity can occur.

## Results

Here we describe the case study results for two autosomal recessive and one X-linked dominant disease models.

### First case study

Three families presented with hyperinsulinemic hypoglycemia and congenital polycystic kidney disease (HIPKD), a rare newly discovered disorder following an autosomal recessive model. Whole-genome linkage analysis in conjunction with haplotype reconstruction hinted towards a compound-heterozygous disease pattern in all cases within a significant locus on chromosome 16 [3].

Exome-capture sequencing of all cases revealed a promoter mutation paired with either a missense or splice site mutation. To recapitulate the results of this study within OVAS, all four cases were inserted into the pipeline of which two were siblings, permitting the use of variant-level filtering. Pedigree overviews as well as runtime settings conforming to those in the supplemental material of the preceding paper are displayed in the analysis interface (Fig. 2).

Each VCF file comprised of approximately 250,000 variants (SNPs and InDels) and were profiled against a gene map at the first annotation step (*Adding Genes*) comprised of exons, donor/acceptor essential splice sites (5 bp), and upstream/downstream promoter regions (500 bp). Reference genes as well as their isoforms were also retained in the analysis.

The prior linkage analysis [3] hinted at a small region of interest (16p13.3-16p13.2 spanning 2.93 Mbp) populated by 11 genes and 40 isoforms, and applying this locus via the *Physical Location Filter* resulted in 99.9% of variants being filtered out.

The *Core Annotation* stage accounted for the vast majority (>80*%*) of the exome-sequenced variants being filtered out in both scenarios, intersecting variants against the gene map (declared previously) in order to remove those that were entirely intergenic or (non-regulatory) intronic. This resulted in approximately 34,700 annotated variants ready for the subsequent filtering modules.

**Fig. 4:**
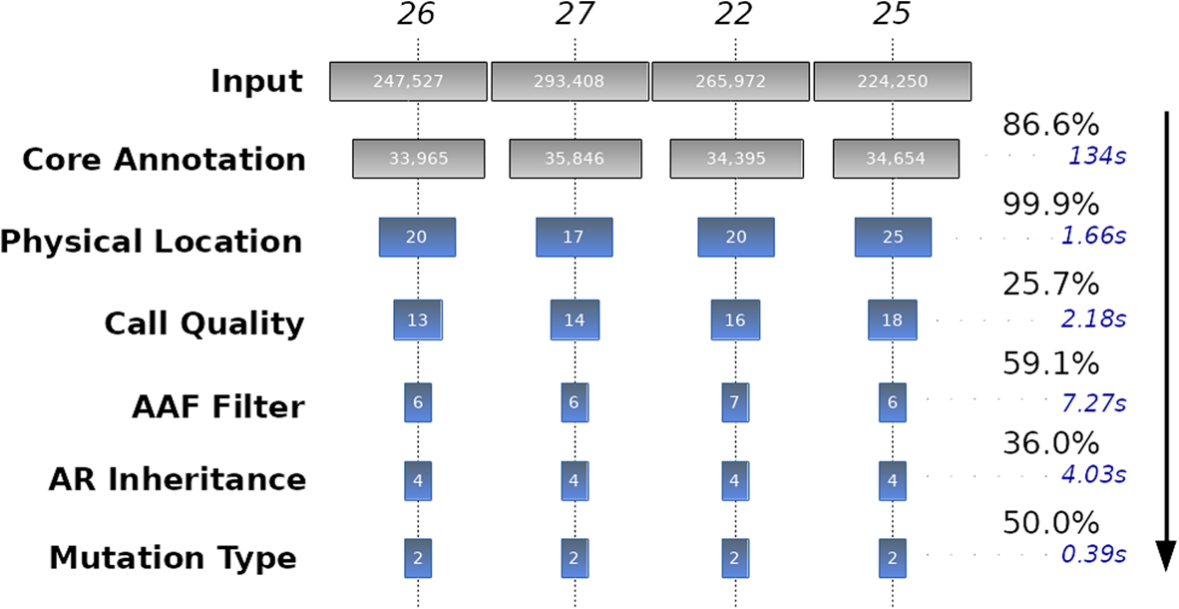
The progression of variants filtered at each subsequent annotation or filtering stage for each of the 4 case VCFs under initial positional filtering. Input and Core Annotation are mandatory steps. Average variant reduction percentages in-between stages are displayed, and average module runtimes are displayed in seconds

The subsequent application of the the *Physical Linkage Filter* reduced the number of variants to less than 25 in each case file (Fig. 4). The *Call Quality Filter* with a threshold of > 20 was applied in accordance to the filtering criterion in the original study, resulting in a 25.7% reduction. The rarity of the phenotype prompted a search for variants not very prevalent in the population, thus the *Alternate Allele Frequency Filter* (AAF) was applied with a threshold of < 1*%*, leaving no more than 10 variants in each case file. The *Autosomal Recessive Inheritance Filter* (AR) then performed identical variant level matching between the two affected siblings, screened against homozygous mutations, and followed compound-heterozygous checking upon all files to produce an overlapping AR gene list.

Truncating under this provided just 4 variants in each file (5 unique in total), and applying the final *Mutation Type Filter* to remove any synonymous mutations resulted in just 2 variants in each file (3 unique in total) that successfully produced a characteristic compound-heterozygous AR inheritance pattern in *PMM2*; *c.-167G* >*T* promoter variant in all, *c.422G* >*A* missense mutation in three of the cases, and a *c.255+1G* >*A* splice site mutation present in one case (Fig. 3).

### Second case study

A single family displaying a phenotype under an X-linked dominant inheritance model. Whole-exome sequencing was performed upon 8 individuals (7 affected, 1 unaffected) with almost 290,000 variants in each VCF file.

As before, the first annotation step filtered out the majority of variants, with an 89.3% reduction due to variants being wholly intergenic/intronic. Significant linkage analysis outlined a narrow region of interest upon chromosome X, which coupled with the *Physical Location Filter* reduced the initial set to just 351 variants (Additional file 1: Figure S1 (top)). A cascade of filters targeting novel non-synonymous mutations under an X-linked dominant scenario (common across affecteds) resulted in a single causative missense variant.

### Third case study

Four siblings were presented from a consanguineous marriage with a nephrotic syndrome segregating in an autosomal recessive fashion. Exome-sequencing was performed on each sibling with an initial targeted set of approximately 70,000 variants. Core annotation accounted for a 65.9% reduction in total variants, and a missense/nonsense *Mutation Type Filter* reduced the initial set to under 11,000 variants (Additional file 1: Figure S2 (bottom)). Due to the rarity of phenotype, the AAF module was utilized to filter for any variants with a frequency less than 0.01 within dbSNP (version 142), vastly reducing the number to a cluster of 878 variants.

Applying the autosomal recessive inheritance module with same variant filtering resulted in just 15 variants common across affecteds only, of which 2 were homozygous in different genes. Additional gene expression annotation was prioritized; with one variant conforming to a standard house-keeping gene expression profile, and the other being the more likely disease-causing variant due to it displaying a strong organ specific expression.

## Discussion

Depending upon the total input variants as well as the number and ordering of modules used, an average initial analysis using any number of modules (excluding alternate allele filtering) for VCF files containing 300,000 variants each, will attribute a total of 2 min per VCF.

There are several limiting steps however, with the largest bottleneck occurring at initial gene annotation stage, which must prime all input variants for downstream filtering through the use of a gene (or exon) map that is dependent upon user parameters. Gene maps for a variety of user parameters already exist as static files in the live environment, but not all use-cases are covered and a new gene map must be generated for custom configurations which can take up to 1 h to retrieve depending on internet speed and proximity to the closest UCSC MySQL mirror.

In the case of general gene map use-cases, the *Adding Genes* annotation step still requires 200 times more processing time than most other modules, and was the sole reason that all annotation modules were re-written in C++ to benefit from a significant performance increase that reduced the module’s processing time from an initial time of 10 min to under 3 min (Table 1).

**Table 1:** Average single-core runtimes of VCF files containing 50,000 variants passing individually through all filters with timings for each Annotation, Filtering, and Extended annotation modules

*Pipeline stage*	*Module name*	*Runtime (seconds)*
	Adding genes	125
Annotation	Adding function	28.7
	Adding Zygosity	0.81
Filtering	Physical location filter	1.02
	Read depth filter	1.26
	Call quality filter	0.93
	AAF filter	143
	Mutation type filter	1.08
	Novel variant filter	1.12
	Same gene filter	22.5
	Same variant filter	26.1
	AD inheritance	0.83
Trait penetrance model	AR inheritance	1.22
	XD inheritance	0.74
	XR inheritance	1.39
	Mosaicism	0.94
Extended annotation	Isoform context	2.28
	Protein context	4.10
	Gene expression	145

Trait Penetrance module timings are based on three VCFs consisting of a parent-offspring trio. Tests were run on a 2GHz dual-core processor with 4GB RAM

The rest of the annotation modules are comparatively much faster, with the functional annotations experiencing mild latency related to disk read speeds when performing repeated byte-offset lookup upon FASTA files. The initial sorting of the variants upon file upload is valuable in this regard due to the higher tendency of adjacent variants to share the same disk cluster and reap paging benefits.

Across subsequent pipeline runs, processing is not repeated for the same data; each module checks whether an input VCF file has already been processed by the current pipeline configuration, and repeatedly iterates through the module ordering until the last processed input set is reached where it can resume processing.

### Case performance

The case analysis completed its run in 10.2 min, with subsequent re-runs upon pre-annotated data completing in under 1 min.

It is not without doubt that the order of filtering modules is important to the analysis, with the *Physical Location Filter* decreasing the runtime of subsequent modules. However this decrease is sub-linear in complexity as shown in Table 1, which displays average individual timings for each module against moderately populated VCF files, showing that runtimes are comparable with the case analysis with the exception of the AAF module.

The AAF module created an noticeable lag of an average of 7.27 s per file in our study. This is owing to the module being subject to some delay in loading pre-computed dbSNP allele frequencies into memory, and due to memory and processing constraints, it must incur this cost for each new chromosome encountered which can create considerable latency in the earlier (larger) chromosomes. The analysis escaped this penalty somewhat by only having to load a single relatively small chromosome into memory.

### Transparency and deployment

The portability of OVAS grants a significant advantage over present-day web-based pipelines by keeping all analyses securely *in situ*, which is greatly beneficial to regions of the world without consistent or active internet in addition to researchers handling personal or private data. The need for accessible offline tools is most present in Africa, where bioinformatical infrastructure and resources are limited [4].

Cloud-based pipelines provide processing power without incurring the hardware cost, but the progression of large whole-genome sequencing data coupled with restricted internet speeds hinder the uptake of these services somewhat as slow transfer speeds ultimately dictate service viability; a factor that is further confounded by the net neutrality debate [13]. Cloud-based analyses also require input data to be uploaded to an external server in order to perform processing, and data ownership after upload is not always retained especially in the case where the work was performed within the cloud [19]. Further, many cloud-services employ non-transparent proprietary methods to reduce the number of false-positives and false-negatives. A common approach is to make use of an internal database or learning algorithm that favours some variants over others based on previous analyses (or a similar training set) [18], resulting in informative variants produced by unquantifiable “black-box” means, creating disparity between the end-user and their analysis.

Transparent filtering methods are likelier to instil greater confidence in the data with the added benefit of customization to better tailor a filter to an analysis in the case of open-source implementations, as with the case of OVAS.

OVAS is bundled within a lightweight Arch Linux environment that contains the pipeline and the web server, static files, and a minimal desktop environment. This is in direct contrast to the more familiar virtualization container platforms such as Docker or Vagrant which provide snapshots of an existing OS, and then must then be run off a virtualization layer that uses more hardware resources during input/output operations than if the OS was run natively [6]. Where virtualization strategies permit wider avenues of deployment, OVAS is specialized to be deployed on bootable mediums and is heavily optimized in this respect in terms of storage and runtime efficiencies which allow it to be run more readily upon more limited hardware by culling any resource-consuming middleware.

Initial development considered the use of pre-existing implicit convention frameworks such as *Snakemake* [12], but a predilection towards coding-flexibility and processing efficiency (especially with respect to extensive use of standard system input/ouput streams) meant that a more unix-driven pipeline framework was required. OVAS uses an over-arching shell-script framework that adheres to good-practice dependency and re-entrancy concepts [15], by managing file dependencies between adjacent modules and by permitting resumeable workflows such that a VCF file will not undergo the same annotation module twice if it has already been processed under the same inputs.

### Comparison to other Bioinformatic utilities

Pabinger et al. [18] surveys over 200 open-source bioinformatic tools, workflows, pipelines, and annotation modules. Workflows and pipelines are similar in function, with the former being a more general-processing framework to aid in the construction of custom pipelines for different data types.

Thirteen pipelines and 9 workflows are compared, of which only 5 cater for VCF files. Most offer commandline access, and most perform variant annotation either by using ANNOVAR [21] for providing a gene and functional context, or annotating metrics based on SNP or sequence analysis (see Additional file 1: Table S1). However, OVAS is the only open-source pipeline that caters for inheritance contexts, and is also the only pipeline with both a commandline and web-interface that is aimed more are bioinformaticians than programmers.

A further 32 distinct variant annotation modules are also compared; 10 which can take VCF files as input but only 4 of which output annotated VCF files (see Additional file 1: Table S2). Other annotators either focus more on upstream genomic formats (FASTA / BAM) or they produce report summaries of the variants; most likely to escape the potential pitfall of the same variant intersecting multiple sites (such as isoforms). OVAS overcomes this limitation by enclosing multiple sites and their related annotations as sideways associative arrays, and treating each site as a single entity when performing filtering later on in the pipeline.

## Conclusions

The self-contained environment provided by OVAS allows researchers to tailor all aspects of their analysis and retain control of their data sets at any phase of processing by means of the transparent open-source modules that comprise the pipeline.

The live environment, paired with the web front-end, provides the additional advantage of abstracting the end-user from the underlying platform specifics by streamlining the input and configuration process, as well as logging active progress descriptions for the current stage of processing, and lastly providing a malleable final report upon all remaining variants discovered complete with dynamic filtering capabilities. The entirety of all uploaded variants are processed first at the gene annotation stage, placing significant strain at the initial stage of the pipeline that is only managed through the use of employing C++ binaries to overcome the performance bottleneck that would otherwise exist with Python/Bash scripts.

The annotation step is crucial, especially for whole-genome sequence data where the vast majority of the variants would be deemed wholly intergenic and would be filtered out as uninformative to the analysis. More common exome-sequencing data typically observe less of a reduction at a much faster processing rate due to the smaller number of total variants, but at the impediment of missing regulatory elements due to lack of coverage. Modules downstream of the annotation stage run trivially, and due to the pipeline’s resume feature which prevents OVAS from processing the same data twice, many subsequent analyses with different module configurations can be run in quick succession after the initial annotation step is complete.

The main inheritance modelling feature provides a unique type of filtering that is not present in any other pipeline, and has a very significant impact in analyses with trios.

OVAS is future-secure due to the inclusion of the background scripts that generated the static data being packaged with the live environment. Updates to the human genome reference, variant databases, and FASTA sequences can be retrieved on demand for platforms with active internet connections. Changes will preserve across successive boots for non-volatile storage mediums such as USB sticks, ideal in deployment scenarios with infrequent or absent internet access. The annotation components will additionally be merged into the Bioconda [7] bioinformatic software distribution for the benefit of the wider bioinformatic community.

## Additional file

Additional file 1 Supplementary Data. (DOCX 104 kb)

## Abbreviations

BAM: Binary alignment map
cDNA: Complementary DNA
C-HET: Compound-Heterozygous
CNV: Copy number variant
FOSS: Free and open source
HET: Heterozygous
HIPKD: Hyperinsulinemic hypoglycemia and polycystic kidney disease
HOM: Homozygous
HTS: High-Throughput sequencing
InDel: Insertion-Deletion
LOD: Logarithm of the odds
OS: Operating system
SNP: Single nucleotide polymorphism
UTR: Untranslateable region
VCF: Variant call format
