# Supplementary material for: OVAS: an open-source variant analysis suite with inheritance modelling
Source: BMC Bioinformatics. 2018 Feb 8;19:46. doi: 10.1186/s12859-018-2030-8 (PMC5806474; doi:10.1186/s12859-018-2030-8)
Supplement: Supplementary file 1 — Supplementary Data. (DOCX 104 kb) [file 12859_2018_2030_MOESM1_ESM.docx]

**Supplementary Data**


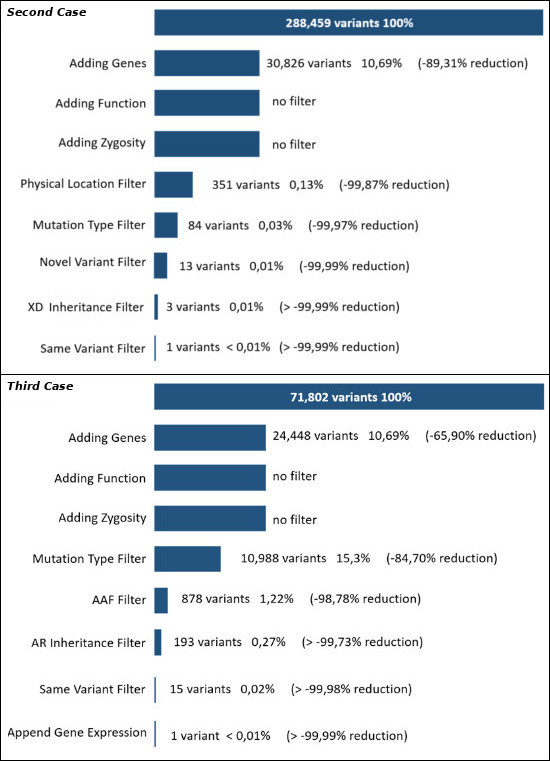
*Figure S1: Annotation and filtering cascade of unique variants across all case VCF input files. Both case studies annotated variants against a hg19 gene map comprised of exons, introns, UTR, splice sites, and 500bp promoter regions. Variants that did not fall within any of these sites were deemed wholly-intergenic and discarded from the analysis. (Top) Second Case study makes use of prior genetic linkage data which highlighted a locus of interest on the X chromosome, and was used in conjunction with the X-linked Dominant Inheritance Filter to reveal 1 causative missense variant. (Bottom) Third Case study looked only for rare non-synonymous coding variants by applying the Mutation Type and Alternate Allele Frequency (AAF) filters in conjunction with an Autosomal Recessive Inheritance Filter, which when combined with additional organ-specific gene expression data, resulted in 1 disease-causing variant.*

**Comparison to other Bioinformatic Tools**

*Table S1: VCF Pipeline and Workflow Comparison*

| **Tool** | **Type** | **Deployment** | **Annotation** | **Functional** | **Filtering** | **Inheritance** | **Interface** | **Cloud** | **End-user** | **Notes** |
| --- | --- | --- | --- | --- | --- | --- | --- | --- | --- | --- |
| **HugeSeq** | Script Pipeline  (Bash/Python) | Desktop | ANNOVAR | ANNOVAR | Y | N | CLI | N | Programmer |  |
| **GATK** | Script Pipeline  (Bash/Python) | Docker | N | N | Y | N | CLI | Y | Programmer | Basic INFO / FORMAT filtering |
| **Galaxy** | Workflow | Docker / Web-server | ANNOVAR  dbSNP/ hapmap | ANNOVAR | Y | N | CLI / Browser | Y and local | BioInf. |  |
| **GenePattern** | Workflow | Desktop / Web-server | Allele Metrics | N | N | N | Browser | Y and local | BioInf. |  |
| **OVAS** | Script Pipeline  (Bash/Python/C++) | OS / Web-server | Y | Y | Y | Y | CLI / Browser | N only  local | BioInf | Bootable |
| **Taverna** | Workflow | Desktop / Web-server | Annotation workflow exists | Y | Y | N | CLI / Browser / App | Y and local | BioInf. | IDE - intensive |

*Table adapted and updated from Pabinger et al.*

*Programs excluded: Genboree (registration issues), Moa (alignment only), Yabi (inaccessible).*

*Table S2: VCF Variant Annotation Comparison*

| **Name** | **SNP** | **VCF I/O** | **Multi-site** | **Gene-Context** | **Functional** | **VCF Filtering** | **CLI** | **GUI** | **End-User** | **Note** |
| --- | --- | --- | --- | --- | --- | --- | --- | --- | --- | --- |
| ANNOVAR | all | both | newline | full | Y | Y | Y | N | Programmer |  |
| AnnTools | all | both | N | full | Y | N | Y | N | Programmer |  |
| F-SNP | SNV | in only | N | full | Y | N | N | Cloud-only | BioInf. | Set of detached databases |
| UCSC Variant Annotator | all | in only | newline | full | Y | N | Y | Cloud-only | BioInf. |  |
| OVAS (Annot) | all | both | Y | full | Y | Y | Y | Browser | BioInf. |  |
| NGS-SNP | SNV | both | - | gene | N | Y | Y | N | Programmer |  |
| SeattleSeq | SNV/Indel | in only | N | full | Y | N | N | Cloud-only | BioInf. |  |

*Table adapted and updated from Pabinger et al.*

**Evaluation of Common Filtering Utilities**

Two different sets of cases were used. The first column describes the annotation/filtering steps that were applied; the next two columns indicate the average number of variants after the respective annotation/filtering steps.

***ANNOVAR***

*Table S3: Annotation/filtering performed via ANNOVAR.*

| **Annotation / Filtering** | **Case 1** | **Case 2** |
| --- | --- | --- |
| Initial No of Variants | 265970 | 288459 |
| Gene-based | 265970 | 265970 |
| Region-based | 567 | 459 |
| Filter based:   - Allele frequency <1% - Prediction tools |  |  |
|  | 102 | 81 |
|  | 0 | 0 |

The following annotation/filtering operations were used:

- *Gene-based*: annotation of the variant that determines position and change at the cDNA and protein level.
- *Region-based*: keeps variants within the linkage region which is defined by the user.
- *Filter-based*: allele frequency filter keeps only rare variants that can be defined by the user and prediction tool filter leaves variants that are only predicted as deleterious.

ANNOVAR removed all the variants of interest from the control projects in the final output due to the final filtering step which predicts the deleteriousness of variants.

***VCFTools***

*Table S4: Filtering performed through the VCFtools pipeline.*

| **Filtering** | **Case 1** | **Case 2** |
| --- | --- | --- |
| Initial no of Variants | 265970 | 288459 |
| Position-filtering | 567 | 459 |
| Allele-filtering <1% | 112 | 95 |
| Comparison-filtering | n/a | 68 |

The following annotation/filtering operations were used:

- *Position-filtering*: keeps variants within the linkage region which is defined by a user.
- *Allele-filtering*: keeps variants that are rare which is defined by a user.
- *Comparison-filtering* (used only for Case 2 since Case 1 did not have any controls): compares variants between affected vs unaffected and filters out those that are present in both.

The VCFtools package has many filtering options however, few are appropriate for finding rare Mendelian variants. As a result, too many variants are left in for the final analysis.

**Implementation (Detailed)**

***Application Suite and Interface***

The pipeline was originally developed in a headless Linux shell environment to be deployed on any Unix-like system that supports Bash, appealing to experienced technicians who can perform their own input validation. However, significant effort was made to include researchers from non-computing backgrounds who could benefit from the rich processing without the cost of additional footwork.

***Web-Front***

To necessitate the uptake of OVAS, a web interface was created to facilitate input validation and pipeline configuration process. The file upload procedure is streamlined by means of a pedigree file which pre-specifies cases (affecteds) and controls (unaffecteds) as well as their relation to one another. Pedigree data is automatically parsed into a table of cases and controls for all detected families, with file upload slots being assigned for each individual. The user is then given the choice to select multiple VCF files which are then mapped automatically to the appropriate individual slots, or separate files can be dragged and dropped if more manual assignment is desired.

The interface extends to display configuration options for each annotation and filtering module whilst uploading occurs in the background. Modules are enabled by expanding check-boxes to display individual module parameters and thresholds that can be overridden by user criterion, examples of which can be shown in Figure 2.

A drop-down box of available penetrance model provides mutually-exclusive model-dependent options to better refine the analysis, such as parent or unaffected sibling-specific filtering. Additional annotation requirements are set (or skipped upon preference) and then the pipeline is run in tandem to the existing input session. In the case of user-termination, re-upload is not necessary for the same analysis as the process will reuse the temporary files from the last session and will not repeat the same work twice, resuming from where it left off. Once complete, the pipeline self-terminates and produces an interactive report of the remaining variants primed for feature presentation/concealment to help pinpoint variants of interest such as those shown in Figure 3.

The pipeline is spawned in a GNU screen session in order to enable process control and resumeability, where snapshots of a session in-process are repeatedly retrieved from the shell process to the web front-end via PHP scripts. UI elements are managed with CSS and minimal JavaScript, with the exception of the interactive report which performs table operations primarily through the latter. The front-end itself is hosted via a minimal lighttpd server, and ongoing OVAS processes can be managed both from the web-interface as well as from the shell provided in the live environment.

***Self-Contained Environment***

The full OVAS suite comprises of the core pipeline processing back-end encapsulated by the web-interface to handle input validation, which is encapsulated once more by a live operating system that handles and provides general file utilities as well as overall startup. Each of these three components exist as separable peripherals, but are optimal in the above configuration by facilitating and abstracting the installation of each through the use of symbolic links and providing constant anchors for static data bundled with the environment.

OVAS runs straight off the X desktop server within a minimal Arch Linux environment, with the OVAS interface autostarted along with a minimal dock for spawning additional applications.

The static data primarily encompasses a variety of gene map configurations from human genome reference version hg18 through to hg38, as well as the raw nucleotide FASTA files for each chromosome specific to the versions, amounting to 15GB of genomic data. Due to the packing process, as well as compression algorithm used in the Squash Filesystem creation process OVAS amounts up to no more 2.7GB. This makes it ideal for bootable mediums such as DVDs and USB sticks, where the latter can preserve data across subsequent sessions.

**Pipeline Modules (Detailed)**

Each module is tasked with the function of separating variants from an input file into two distinct output VCF groups of "filtered" and "discarded"; with the former group being passed into the next module, and the latter being halted at the current point of processing to be stored for potential debugging purposes. The discard process at each module lends a progressive performance increase in the processing speed of each subsequent module due to the input being only a subset of the input that came before it, whilst still retaining the aggregate total of discarded variants at each step.

**Pre-processing**

All VCF files immediately undergo initial preparation upon file upload from the web interface, where a background shell script renames the files to better emulate their pedigree counterparts, and asserts that all variants are in correct order following a chromosome:position sorting key.

**Core Annotation**

The annotation stages of the pipeline then prime the variants with relevant metadata that will then be filtered against user-criterion throughout the rest of the pipeline. The annotation stage is the only mandatory stage of the pipeline, and a great portion of filtering occurs at these stages too, with up to 90\% of true negatives being discarded. As a result of the large demand placed upon the modules at this stage, they were written in the C++ in order to reduce time and memory constraints on low-end platforms. The stage is split into three modules (in order of processing):

***Adding Genes***

Appends a gene-context to the variants under a user-configured level of detail at the gene/intergenic junction or the exon/intron/splice/UTR sub-divisions, including isoforms. Regulatory variants further up or downstream of UTR can be specified by defining custom margins of enclosement, and wholly intergenic regions are discarded by default (though can be kept upon user preference).}

***Adding Function***

Applies functional annotation upon the variants processed in the previous step; performing a cDNA lookup of where a variant falls within the coding portions of the gene in order to predict the type of mutation (missense, synonymous, or non-synonymous) at the codon and subsequent amino-acid level. Anti-sense encoded genes are handled accordingly, and for insertion/deletion (indels) variants the module performs the required addition/subtractions across a consistent reading frame to discern the mutation.

***Adding Zygosity***

Addresses a confidence issue in with pre-processed variants, where heterozygosity and homozygosity would be assigned based on post-quality filtering metrics. This module sets zygosity by nucleotide base-count alone, and determines HET/HOM assignments based upon a user-set frequency threshold (default <0.65). Once fully annotated, the resultant output VCFs are ready to be processed by the filtering modules.

**Filtering Modules**

The filtering modules consist of a series of Python (v2.7) scripts designed to parse these fields with the aim of minimizing the need for any mapping or additional pass-throughs. A variant line in a VCF file describes the eight mandatory fields grouped into three distinct categories (in order of filtering complexity):

***Variant Properties***

The first five mandatory columns (chromosome, physical position (base-pairs), variant-marker identifier, reference allele, and alternate alleles) are processed by two main modules: *Physical Location Filter*, which parses chromosome and physical position to keep variants inclusive to user-set loci ranges; and *Novel Variant Filter* which discards all variants with pre-existing identifiers (i.e., not ‘.’).

***Variant Metadata***

Here, the variant call information field (INFO) is processed, which consists of variant-calling properties summarizing the FASTA strand pileup bisected by the variant. The INFO field alludes to the quality of the sample data, but not to the sample data itself, enabling for fast single-pass processing by the following four modules: *Read Depth Filter* and *Call Quality Filter*, which both discard variants falling below a user-set limit, the former upon the number of FASTA reads aligned at that position, and the latter upon the variant calling score value; *Mutation Type Filter*, which makes use of functional annotations in order to filter single variants based upon user-set requirements of including any (multiple) of missense, nonsense, and synonymous mutation types; and finally *Same Gene Filter* and *Same Variant Filter*, that act upon multiple VCFs and produce a common output set of variants that reside in either the same gene(s) or share identical variants respectively.

***Sample Data***

The sample format field (FORMAT) structures the presentation that all subsequent sample data conform to, and must be parsed before handling of the actual data. The *Alternate Allele Frequency* module scans the sample data in order ascertain the absolute frequencies of the alternate allele(s) in the population whilst removing variants with frequencies exceeding user-defined upper/lower-bound thresholds.

**Inheritance Filtering**

(clarified in main text)

**Extended Annotation**

The last stage of pipeline constitutes a small subset of variants which have successfully passed through the main filtering stages and require finer analysis which is enabled by providing an even greater context to compare the variants. Additional annotation relates to the downstream effects of said variants such as structure, function, and expression.

*Isoform Context -* Translates gene isoforms into their RefSeq nomenclature counterparts.

*Protein Context -* Assigns protein annotation information from UniProt sources to assign information related protein domain.

*Gene Expression -* Organ and tissue-specific data from the Encode GNF Atlas2 database is provided along with expression ratios which can be further filtered against user-specified limits.
